# Dopaminergic modulation of performance monitoring in Parkinson’s disease: An event-related potential study

**DOI:** 10.1038/srep41222

**Published:** 2017-01-24

**Authors:** Caroline Seer, Florian Lange, Sebastian Loens, Florian Wegner, Christoph Schrader, Dirk Dressler, Reinhard Dengler, Bruno Kopp

**Affiliations:** 1 Department of Neurology, Hannover Medical School, Hannover, Germany.

## Abstract

Monitoring one’s actions is essential for goal-directed performance. In the event-related potential (ERP), errors are followed by fronto-centrally distributed negativities. These error(-related) negativity (N_e_/ERN) amplitudes are often found to be attenuated in patients with Parkinson’s disease (PD) compared to healthy controls (HC). Although N_e_/ERN has been proposed to be related to dopaminergic neuronal activity, previous research did not find evidence for effects of dopaminergic medication on N_e_/ERN amplitudes in PD. We examined 13 PD patients “on” and “off” dopaminergic medication. Their response-locked ERP amplitudes (obtained on correct [N_c_/CRN] and error [N_e_/ERN] trials of a flanker task) were compared to those of 13 HC who were tested twice as well, without receiving dopaminergic medication. While PD patients committed more errors than HC, error rates were not significantly modulated by dopaminergic medication. PD patients showed reduced N_e_/ERN amplitudes relative to HC; however, this attenuation of response-locked ERP amplitudes was not specific to errors in this study. PD-related attenuation of response-locked ERP amplitudes was most pronounced when PD patients were on medication. These results suggest overdosing of dopaminergic pathways that are relatively spared in PD, but that are related to the generation of the N_e_/ERN, notably pathways targeted on the medial prefrontal cortex.

Parkinson’s disease (PD) is a common neurodegenerative disease, with an incidence of 8–18 per 100,000 person-years^1^. Idiopathic PD is hallmarked by the progressive loss of dopaminergic neurons in the substantia nigra pars compacta, resulting in striatal dopamine depletion. Besides the cardinal motor symptoms (bradykinesia, resting tremor, rigidity, and postural instability), cognitive impairment and executive dysfunction are frequently observed in PD^2^,^3^.

Monitoring one’s own performance is a central aspect of executive functioning. When having committed an error, subsequent performance adjustments may be necessary to ensure that a pursued goal can still be achieved. Hence, successful goal-directed actions require the detection and evaluation of errors^4^,^5^. In the event-related potential (ERP)^6^, erroneous actions are typically followed by a fronto-centrally distributed negativity^7^,^8^. This error(-related) negativity (N_e_/ERN) is considered as a neural correlate of performance monitoring^4^. It is probably generated in the anterior cingulate cortex (ACC; more precisely, in the anterior midcingulate cortex)^4^ and has been proposed to be related to signalling from the mesencephalic dopamine system to the ACC^9^ (but see ref. 5 for critical discussion). The amplitude of N_e_/ERN is typically found to be reduced (i.e., less negative) in patients with PD compared to healthy controls^10^,^11^,^12^,^13^,^14^,^15^,^16^ (but see refs 17 and 18; a detailed overview is provided by ref. 19; see also ref. 20), possibly as a result of the PD-related dopamine depletion.

The assessment of patients with PD offers the possibility to investigate the influence of dopaminergic medication on ERP correlates of cognitive functions in a clinically relevant context. For instance, patients can be examined under their usual doses of dopaminergic medication (“on”) and after withdrawal from that medication (“off”). Withdrawal from dopaminergic medication, particularly levodopa, is comparably uncomplicated due to the short half-life of this drug^21^. It is also possible to assess the same individuals both on and off medication in a within-subjects design. These intraindividual comparisons of on and off medication states are particularly powerful due to the minimization of unsystematic interindividual variance.

To date, only one study has compared N_e_/ERN amplitudes in PD patients in a within-subjects design between on and off medication states, and it did not find a difference between these two conditions^15^. However, the patients included in that study were examined relatively early in the course of the disease, with an average disease duration of 3.2 years. The average symptom severity (assessed off medication with the Unified Parkinson’s Disease Rating Scale-III [UPDRS-III]) was comparably mild (14.8, cf. ref. 19). It is thus possible that the doses of dopaminergic medication that were administered to these patients were not sufficient to exert a detectable effect on the N_e_/ERN amplitude.

The present study contributes to the literature by examining the effect of dopaminergic medication on N_e_/ERN amplitude in a sample of patients with a longer average disease duration (11.3 years) and more severe motor symptoms (UPDRS-III score off medication: 26.3). We expected to replicate the well-established attenuation of N_e_/ERN amplitudes in PD patients. Furthermore, we investigated to which extent this attenuation would be affected by dopaminergic medication.

## Methods

### Participants

Twenty-two non-demented inpatients and outpatients with idiopathic PD were recruited from the Department of Neurology, Hannover Medical School. The diagnosis was confirmed by an experienced attending neurologist (Ch.S., D.D., F.W., R.D.). Patients were not included when they had any severe neurological or psychiatric condition other than PD, or a history of neurosurgical therapy. All participants in the study had scored a minimum of 23 points on the Montreal Cognitive Assessment (MoCA)^22^.

Patients were examined twice: once under their usual medication (“on”) and once after withdrawal (“off”) from dopaminergic medication. Twelve patients were examined off medication in their first session and on medication in their second session, whereas 10 patients were examined on medication in their first session and off medication in their second session. One patient was excluded after testing due to extremely prolonged reaction times (>3 SD of the sample mean). For older adults, good internal consistency of N_e_/ERN amplitudes can be achieved based on eight trials^23^. Therefore, individuals for whom less than eight artefact-free error trials were available (i.e., another eight patients) were excluded from further analyses. In the final patient sample (*N* = 13; 3 females), the median Hoehn-and-Yahr stage was 3 (range: 2–4). The average disease duration was 11.31 years (*SD* = 7.09). Table 1 displays further sociodemographic information and clinical details of the patients. The UPDRS-III score was used to assess motor functions in the patients on and off medication. UPDRS scores were obtained from the patients’ recent medical records; this measure could not be obtained from one patient. Five patients (1 female) were first examined off medication, and eight patients (2 females) were first examined on medication. The median time between the two sessions in the PD group was 2 days (range: 1–29). The median duration of withdrawal from medication before the off-medication session was 14 hours (range: 4–142). The average levodopa equivalent daily dose of antiparkinsonian medication that was administered to the patients at the time of the on-medication session was calculated using the conversion factors provided by ref. 24 and amounted to 1,015 mg/d (*SD* = 509). Table 2 displays the doses of antiparkinsonian medication for each patient at the time of the on-medication session. The present study aimed at analysing the effects of dopaminergic medication by comparing differences between on and off states. Therefore, it was ensured that the two testing sessions for the PD patients differed with regard to whether or not dopaminergic medication had been administered to the patients prior to testing, but no further objective criterion was applied to determine whether they were in a maximal clinical on or off state during the time of testing.

Thirty-five control participants were recruited by posters distributed throughout the city of Hannover, Germany, and word-of-mouth advertising. None of the healthy controls (HC) was diagnosed with severe neurological or psychiatric conditions. One HC was excluded because of having scored lower than 23 on the MoCA.

No medication was administered to the HC as a part of the study, but they were examined twice as well to control for potential effects induced by repeated testing. One HC was excluded after testing due to extremely prolonged reaction times (>3 SD of the sample mean). Another 16 HC were excluded from further analyses, because less than eight artefact-free error trials were available for these individuals for at least one testing session. From the remaining 17 HC, a subset of *N* = 13 HC (3 females) was selected to form the final sample such that the gender distribution was matched and the mean age was as similar as possible between the PD and the HC group. To allow controlling for order effects of testing sessions in patients, every HC was randomly assigned either to the order “first session off—second session on” (*n* = 5; 1 female) or to the order “first session on—second session off” (*n* = 8; 2 females), with the constraint that these groups should match the corresponding patient subgroups in group size and gender distribution. The median delay between the two sessions in the HC group was 1 day (range: 1–11). The two testing sessions took place at the same time of day for any individual participant, with the exception of one HC and three PD patients. The median deviation between the daily starting times of the two testing sessions were 20 and 54 minutes for HC and PD, respectively.

All participants were offered a compensation of 50 €. The study was approved by the ethics committee of Hannover Medical School (vote number: 6589), and all procedures were carried out in accordance with the approved guidelines. All participants gave written informed consent in accordance with the Declaration of Helsinki.

### Materials and procedure

**Figure 1:**
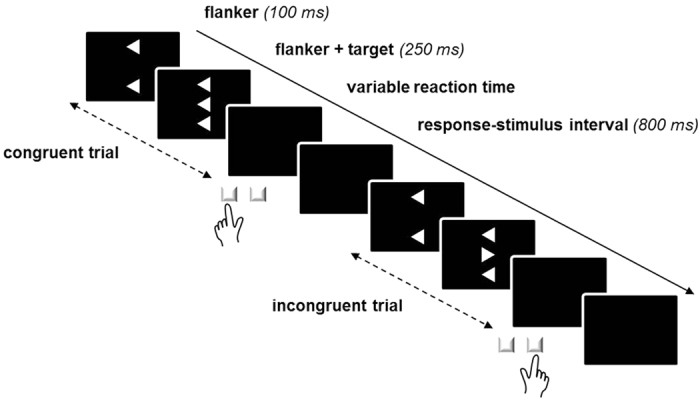
Exemplary trial sequence on the Eriksen flanker task. On every trial, three vertically arranged arrowheads were presented, each either pointing to the left or to the right. Participants were asked to respond to the central (“target”) arrowhead by pressing a spatially compatible key while ignoring the distracting “flanker” arrowheads above and below the target arrowhead. On congruent trials, flanker and target arrowheads point to the same direction, whereas on incongruent trials, flanker and target arrowheads point to opposite directions. Flankers were set to precede the targets by 100 ms. The whole stimulus array (flanker + target) remained on screen for 250 ms. When a motor response (i.e., a key press) had occurred, the next trial was presented after 800 ms.

All participants completed 432 trials (divided into four blocks and preceded by 12 practice trials) of a computerized version of the Eriksen flanker task^25^,^26^ (Fig. 1). Stimulus material was run by Presentation^®^ (Neurobehavioral Systems, Albany, CA). Stimuli were presented against a black background on a 24-inch flat screen (Eizo EV2416W, Hakusan, Ishikawa, Japan). Responses were collected using a Cedrus^®^ response pad (RB-830, Cedrus, San Pedro, CA).

Every stimulus consisted of three white arrowheads (subtending a visual angle of 4.5° × 1.2° at 1.20 m viewing distance, with vertical distances of 0.4° between the arrowheads), each pointing to the left or to the right. Arrowheads were arranged vertically such that the two outer arrowheads (“flanker”) either pointed to the same (congruent) or to the opposite (incongruent) direction compared to the central arrowhead (“target”). Targets were presented for 250 ms after an onset delay relative to the flankers that was set to 100 ms. Flankers remained on the screen until target offset. Participants were asked to ignore the flankers and respond to the targets by pressing a spatially compatible key (i.e., a left-hand key press on the leftmost key on the response pad was required whenever the target pointed to the left side, and a right-hand key press on the rightmost key on the response pad was required whenever the target pointed to the right side).

All participants were asked to complete a battery of psychometric questionnaires; the results are displayed in Table 1. A German vocabulary test (Wortschatztest, WST)^27^ was used to estimate premorbid crystallized intelligence.

### Electrophysiological recording

Continuous electroencephalogram (EEG) was recorded with a BrainAmp amplifier and 30 active Ag-AgCl electrodes (Brain Products, Gilching, Germany) mounted on an actiCap (EASYCAP, Herrsching, Germany) according to the international 10–20 system montage. BrainVision Recorder 1.2 (Brain Products, Gilching, Germany) was used; the sampling rate was 250 Hz. Electrode impedance was kept below 10 kΩ. Electrodes were referenced to FCz electrode. To monitor ocular artefacts, vertical (vEOG) and horizontal (hEOG) electrooculogram were recorded with two electrodes positioned at the suborbital ridge and the external ocular canthus of the right eye, respectively.

### Data analysis

Data were analysed using IBM SPSS 23.0 and 24.0. The significance level was set to 0.05. Effect sizes were calculated as 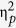 (as implemented in SPSS) and Cohen’s *d* (according to the supplement [version 3.4] provided by ref. 28).

#### Behavioural data

Reaction time (RT) and error rate (ER) were calculated separately for congruent and incongruent trials. RTs were obtained by computing the median response latency separately for correctly completed and error trials. Responses that were registered earlier than 100 ms or later than 2000 ms after target onset were excluded. ERs were calculated as the proportion of erroneous responses (i.e., a button press corresponding to the opposite direction of the target). Both RTs and ERs were only calculated for trials preceded by correctly completed trials to rule out the influence of processes induced by erroneous responding on the previous trial (e.g., post-error slowing^29^, errors induced by irritation about having committed an error on the previous trial).

Relationships between the behavioural outcome measures RT (on correctly completed trials) and ER and the variables congruency, medication, and PD were analysed by repeated-measurement ANOVAs with Session (off, on) and Congruency (congruent, incongruent) as within-subjects factors, and Group (PD, HC) as between-subjects factor. We also compared RTs on error trials to RTs on correctly completed trials using a Session (off, on) × Correctness (correct response, error) × Group (PD, HC) repeated-measurement ANOVA. The factor Congruency was not included in this analysis because errors rarely occur on congruent trials. For PD patients, the Session factor indicated the actual medication status (i.e., whether the patients had taken their usual dose of medication [on], or were tested after withdrawal from medication [off]). For HC, the Session factor was merely used to assign control participants to either the “first session off—second session on” (*n* = 5) or the “first session on—second session off” (*n* = 8) order to allow controlling for order effects when comparing PD patients to HC.

#### Electrophysiological data

EEG data were evaluated using BrainVision Analyzer 2.0 (Brain Products, Gilching, Germany). The data were filtered with a high-pass filter of 0.1 Hz (24 dB/oct) and a low-pass filter of 70 Hz (24 dB/oct), which were chosen to minimally distort the data. High-frequency artefacts introduced by electrical noise were filtered out with an additional notch filter (50 Hz). Data were screened for remaining artefacts (voltage step >75 μV/ms; activity <0.5 μV/100 ms) and subjected to an independent component analysis^30^ for correction of ocular, muscular, and cardiac artefacts. As errors rarely occur on congruent trials, response-locked ERPs were compared for correct responses and errors on incongruent trials only. Data obtained on correctly and incorrectly completed trials were separately segmented into epochs relative to response onset and baseline-corrected (−200 to 0 ms). Residual artefacts (value difference >150 μV/200 ms; amplitude <−100 μV or >100 μV) were rejected. After averaging and re-referencing to a common average reference, N_e_/ERN amplitude and the amplitude of the corresponding negativity on (incongruent) correct trials (“correct(-related) negativity”, N_c_/CRN^31^,^32^) were quantified at FCz electrode as the first negative peak following the overt erroneous and correct response, respectively. Supplementary Table S1 displays the numbers of trials included in the ERP analyses.

Relationships between response-locked ERPs and the variables correctness, medication, and PD were analysed by repeated-measurement ANOVAs with Session (off, on) and Correctness (correct response [N_c_/CRN], error [N_e_/ERN]) as within-subject factors, and Group (PD, HC) as between-subjects factor. Recall that the Session factor indicated the actual medication status for the PD patients, whereas for HC, it was merely used to assign participants to the “first session off—second session on” or the “first session on—second session off” order. If dopaminergic medication did indeed affect response-synchronized ERP amplitudes, a Session effect should emerge in PD patients, but not in HC (in this group, the two levels of Session do not reflect different levels of medication), i.e., the interaction of Session and Group should be significant. If the effect of medication on ERP amplitudes in PD was error-specific, the interaction between Session, Correctness, and Group should be significant.

We additionally tested our main questions directly. Specifically, to compare whether N_e_/ERN amplitudes are smaller in PD than in HC, we compared N_e_/ERN amplitudes (averaged across the two sessions) between these groups using an independent samples *t*-test. To directly test the effect of medication on N_e_/ERN amplitudes in PD, we used a paired samples *t*-test.

#### Correlation analyses

We also explored the relations between N_e_/ERN amplitudes and clinical/psychological characteristics of the participants. To this end, Spearman-Brown correlation coefficients (*r*_*s*_) were calculated.

## Results

Patients’ UPDRS-III scores (with lower values indicating better motor performance) were significantly lower on (17.42) compared to off (26.33) dopaminergic medication. Thus, the pharmacological treatment was sufficient to exert beneficial effects on motor functions, *t*(11) = −5.62, *p* < 0.001, *d*_*z*_ = −1.62.

### Behavioural data

Table 3 displays descriptive information for RTs and ERs in PD and HC. RTs on correctly completed trials were modulated by Congruency, *F*(1, 24) = 221.38, *p* < 0.001, 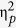 = 0.90, with longer RTs on incongruent (503 ms) compared to congruent (424 ms) trials. RTs on correctly completed trials were not significantly affected by either Session or Group, and the interactions involving Session and Group were not significant (all *F* < 2.37, all *p* > 0.05).

Comparisons between correctly completed and error trials revealed that RTs were significantly shorter on (incongruent) error trials (396 ms) compared to correctly completed (incongruent) trials (503 ms), as indicated by a significant main effect of Correctness, *F*(1, 24) = 25.94, *p* < 0.001, 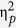 = 0.52. No other main effect or interaction was significant (all *F* < 3.63, all *p* > 0.05).

ERs were modulated by Congruency, *F*(1, 24) = 30.53, *p* < 0.001, 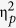 = 0.56, with higher ERs on incongruent (16%) compared to congruent (5%) trials. PD patients (15%) committed significantly more errors than HC (6%) as indicated by a significant main effect of Group, *F*(1, 24) = 8.35, *p* = 0.008, 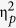 = 0.26. ERs were not significantly influenced by Session, and no interaction involving Session or Group was significant (all *F* < 1.11, all *p* > 0.05).

### Electrophysiological data

**Figure 2:**
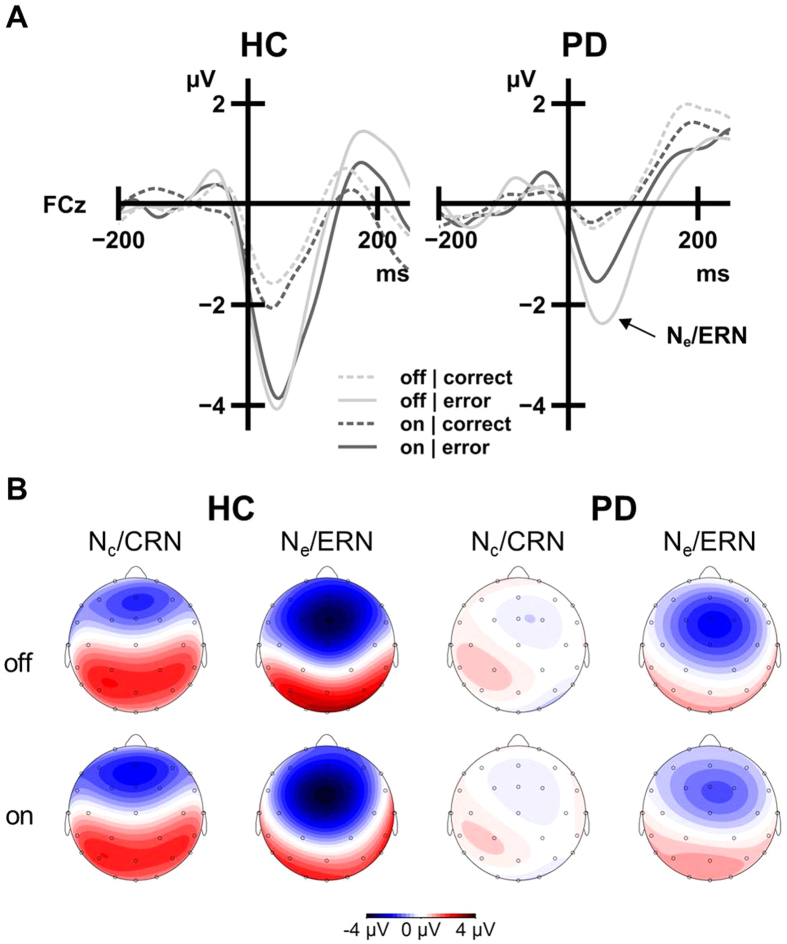
Session effects on response-synchronized ERPs. N_e_/ERN amplitudes were attenuated in the “on-medication” state (dark grey) compared to the “off-medication” state (light grey) in PD patients (right panels). HC (left panels) did not receive dopaminergic medication, and the ERP amplitudes did not differ between the sessions. (**A**) Response-synchronized grand average ERP activity following correct responses (N_c_/CRN, dashed lines) and errors (N_e_/ERN, solid lines). ERPs were low-pass filtered (12 Hz, 24 dB/oct) for display purposes only. (**B**) Topographical information for N_c_/CRN and N_e_/ERN (at grand average peak latency) for the two sessions in HC and PD.

Figure 2 depicts the response-synchronized grand-average ERP activity (see Supplementary Figure S1 for stimulus-synchronized grand-average ERPs). Analysis of these ERPs (Fig. 2A; Supplementary Table S2) revealed the typical main effect of Correctness, with significantly larger amplitudes following errors (N_e_/ERN: −3.95 μV) compared to correct trials (N_c_/CRN: −1.66 μV), *F*(1, 24) = 23.94, *p* < 0.001, 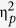 = 0.50. Amplitudes were generally attenuated in PD patients (−1.90 μV) compared to HC (−3.71 μV), as indicated by a significant main effect of Group, *F*(1, 24) = 7.72, *p* = 0.010, 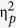 = 0.24. The interaction between Correctness and Group was not significant, *F*(1, 24) = 1.18, *p* = 0.289, 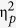 = 0.05, hence the PD-related amplitude attenuation was not specific to error trials. Amplitudes were modulated by a Session by Group interaction, *F*(1, 24) = 6.36, *p* = 0.019, 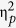 = 0.21, indicating differential effects of Session (representing medication effects in PD but not in HC) for PD and HC. No other main effect or interaction was significant (all *F* < 1.67, all *p* > 0.05).

For follow-up analyses, response-locked ERP amplitudes were collapsed across correct and incorrect trials by averaging N_c_/CRN and N_e_/ERN amplitudes separately for the off and the on session. The resulting response-locked ERP amplitudes were then compared between PD patients and HC using independent samples *t*-tests. In the off session, PD patients (−2.18 μV) showed numerically smaller amplitudes than HC (−3.54 μV), but this Group difference was not statistically significant, *t*(24) = 1.96, *p* = 0.061, *d*_*s*_ = 0.77. In contrast, response-locked ERP amplitudes were significantly attenuated in PD patients (−1.62 μV) as compared to HC (−3.87 μV) in the on session, *t*(24) = 3.45, *p* = 0.002, *d*_*s*_ = 1.35. Taken together, response-locked ERP amplitudes were attenuated in PD patients compared to HC. These alterations were particularly circumscribed when the PD patients were under dopaminergic medication.

To directly answer our first initial question whether N_e_/ERN amplitudes are attenuated in PD patients compared to HC, an independent samples *t-*test of N_e_/ERN amplitudes (collapsed across both sessions) was calculated. It revealed a difference between PD patients (−2.79 μV) and HC (−5.10 μV), *t*(24) = 2.55, *p* = 0.018, *d*_*s*_ = 1.00. Our second main question was whether these N_e_/ERN amplitudes are altered by dopaminergic medication in PD. We used a paired samples *t*-test to compare N_e_/ERN amplitudes off vs. on medication for PD patients only. This analysis revealed that N_e_/ERN amplitudes in PD were smaller on (−2.33 μV) compared to off (−3.26 μV) dopaminergic medication, *t*(12) = −2.78, *p* = 0.017, *d*_*z*_ = −0.77. Taken together, these results suggest that dopaminergic medication in doses that provide relief from motor symptoms may aggravate the well-established N_e_/ERN amplitude attenuation in PD.

### Correlation analyses

Table 4 displays the results of exploratory analyses of potential relations between N_e_/ERN amplitudes and clinical and psychological characteristics of the participants.

## Discussion

We compared response-locked ERP waveforms observed during the commission of errors in a flanker task in PD patients when they were on and off dopaminergic medication. N_e_/ERN amplitudes were reduced in PD patients as compared to healthy control participants, and this amplitude reduction appears to be most pronounced when PD patients are under dopaminergic medication. However, it is important to consider that in our study—in contrast to previous studies^14^,^15^,^16^—the PD-related reduction of response-locked amplitudes was not error-specific (i.e., the Correctness × Group interaction was not significant). Therefore, it cannot be concluded from our data alone that the N_e_/ERN is specifically reduced in PD. However, since a large body of previous research has already established a PD-related attenuation of N_e_/ERN amplitudes^19^, it is most likely that the sample size in our study was not sufficiently large to detect the Correctness × Group interaction. Although the same limitation also applies to medication effects on N_e_/ERN amplitudes, our data indicate that dopaminergic medication aggravates, rather than counteracts, this PD-related attenuation of response-locked ERP amplitudes. The error-specificity of the dopamine effect in PD remains to be corroborated in future studies with larger samples, particularly in light of previous studies that did not find a dopamine effect on N_e_/ERN amplitudes altogether^13^,^15^ (see below).

Response-locked ERP amplitudes in the latency area of the N_e_/ERN have often been found to be reduced in PD^19^. The role of dopamine in the PD-associated N_e_/ERN amplitude reduction has been a subject of discussion^20^,^33^, and this study is the first to show an effect of dopaminergic medication on response-locked ERP amplitudes in PD. According to the influential model proposed by Holroyd and Coles^9^, errors are followed by phasic decreases in dopamine firing from the mesencephalic dopamine system to the ACC, and the N_e_/ERN is thought to be generated by the resulting increase in ACC activity (due to the disinhibition of ACC neurons by the reduced dopaminergic input). Hence, changes in dopamine levels after administration of dopaminergic medication should be associated with N_e_/ERN alterations. The results presented here are partly consistent with Holroyd and Coles’ theory in that they support a relation between the dopaminergic medication status in PD and N_e_/ERN amplitudes. Note however that this framework cannot explain why the dopamine effect on response-locked ERP amplitudes was not specific to errors in the present study.

**Figure 3:**
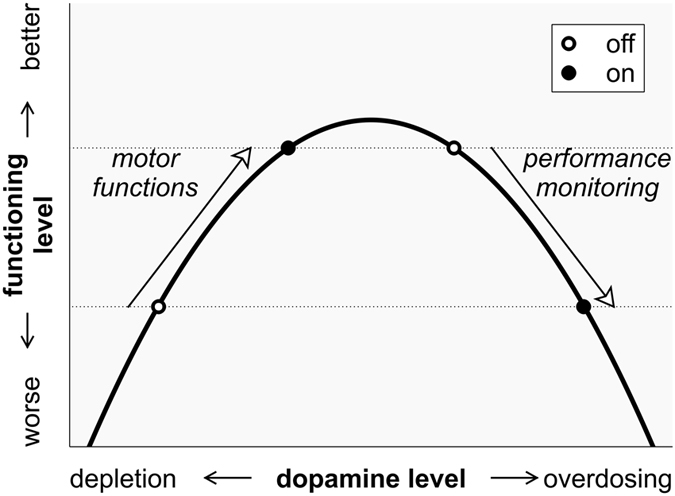
Illustration of the proposed inverted U-shaped relation between dopamine levels and functions that are associated with dopaminergic systems^35^. Both too low (depletion) and too high (overdosing) levels of dopamine may impair motor and/or cognitive performance. Therapeutic administration of dopaminergic medication is usually titrated to optimize motor functions that are related to dopaminergic pathways involving the dorsal striatum. These functions are ameliorated in the sense that they are shifted from a relatively low functioning level (lower dotted horizontal line) to a higher one (upper dotted horizontal line) by substitution of dopamine (left arrow). The necessary dopamine doses may simultaneously impair other (e.g., cognitive) functions that are associated with brain areas which are less affected by the disease. These functions are impaired in the sense that they are shifted from a relatively high functioning level (upper dotted horizontal line) to a lower one (lower dotted horizontal line) by the systemic administration of dopaminergic medication (right arrow).

A medication-related reduction of N_e_/ERN amplitudes (rather than an enhancement) would be in accordance with the dopamine overdose hypothesis^34^,^35^. This framework assumes that medium (optimal) levels of dopamine are required by both motor and cognitive functions that are related to dopaminergic systems, and that both too low (depletion) and too high (overdosing) levels of dopamine may have detrimental effects^34^,^35^,^36^. Figure 3 depicts the resulting inverted U-shaped relation between both motor and cognitive performance and dopamine levels. The loss of dopaminergic neurons in PD and the associated dopamine depletion predominantly affect the dorsal striatum (via the nigrostriatal dopaminergic pathway), leaving other dopaminergic pathways, such as mesolimbic (projecting from the ventral tegmental area [VTA] to the ventral striatum) and mesocortical (projecting from the VTA to cortical areas) pathways, relatively spared^37^. Dopamine depletion of the dorsal striatum results in the prominent motor impairments, and therapeutic administration of dopaminergic medication is usually titrated to remedy these motor symptoms. Systemically administered dopaminergic medication has also been proposed to simultaneously overdose less affected dopaminergic pathways^34^,^35^,^36^,^37^,^38^,^39^,^40^. Along with the motor functions, those cognitive functions that are mainly associated with the dorsal striatum appear to benefit from dopaminergic medication. In contrast, cognitive functions that are mainly associated with other dopaminergic pathways may be impaired through overdosing induced by dopaminergic treatment^34^,^35^,^36^,^37^,^38^,^39^,^40^. Performance monitoring has been shown to be associated with activity of the ACC, where the N_e_/ERN is probably generated^4^. The ACC is closely related to ventral striatal regions via a fronto-striatal circuit (“anterior cingulate circuit” or “limbic circuit”) and to the VTA via the mesocortical pathway. These dopaminergic pathways are not primarily affected by the PD-related loss of dopaminergic neurons and may therefore be prone to dopamine overdosing^37^,^38^,^39^,^40^. The main result of the present study, i.e., an attenuating effect of dopaminergic medication on response-locked ERP amplitudes in PD patients, might have resulted from the overdosing of brain circuits that are involved in N_e_/ERN generation, such as the anterior cingulate circuit, potentially by overdosing the mesolimbic and/or mesocortical pathway (recall however that the amplitude reduction in PD was not specific to error trials in this study). It is worthy of note that this dopamine overdosing model is typically used to explain dopamine-associated performance decrements in populations of PD patients early in the course of the disease, when the ventral striatum is less affected than the dorsal striatum^37^. This model can only be used to interpret data obtained from PD patients at later disease stages under the assumption that an imbalance between dopaminergic transmission in dorsal and ventral striatal areas remains throughout the course of the disease. It needs to be determined by further studies how diminished response-locked ERP amplitudes in general or N_e_/ERN amplitudes in particular relate to altered dopaminergic neurotransmission in early and in later PD stages.

Our results differ from those reported by Willemssen *et al*.^15^. These authors did not find an influence of dopaminergic medication on N_e_/ERN amplitude in a sample of PD patients (*N* = 18) who were tested both on and off medication. However, the patients included in their study had a shorter disease duration and showed less severe motor symptoms compared to the patients included here. Correspondingly, the doses of dopaminergic medication that they received may have been too low to exert detectable effects on the N_e_/ERN amplitude. Table 1 provided by Willemssen *et al*.^15^ allows for a rough estimate of the individual levodopa equivalent daily dose (LEDD) using the conversion factors given in ref. 24, and therefore enable an approximate between-study comparison of the LEDDs. Indeed, the doses of dopaminergic medication in Willemssen *et al*.’s^15^ study were about 40% lower (601 mg/d) compared to those in the present study (1,015 mg/d). Another study by Stemmer *et al*.^13^ analysed the effects of dopaminergic medication in a between-subjects design, comparing a sample of medicated PD patients (*N* = 9) with a sample of drug-naive PD patients (*N* = 9). These authors did not report a difference between these two PD groups. Hence, in contrast to this study, which provides evidence for a dopamine-related reduction of response-locked ERP amplitudes in PD, two other studies addressing a similar question did not find such medication effects. Although these two studies could be taken to indicate that the effect of dopaminergic medication on N_e_/ERN amplitudes in PD is negligible, this conclusion cannot be drawn based on the absence of significant effects (particularly considering the small sample size in Stemmer *et al*.’s study). Aggregating the results of the existing studies with those of future work will help to estimate the size of these medication effects and determine their meaningfulness.

While dopamine overdosing of pathways associated with N_e_/ERN generation could account for the consistently found amplitude reduction in medicated PD patients^10^,^11^,^12^,^13^, it cannot explain similar findings of reduced N_e_/ERN amplitudes in drug-naive PD patients^13^,^14^,^16^ and PD patients who were tested off dopaminergic medication^14^,^15^. This implies that dopamine overdosing phenomena are unlikely to be the only factor contributing to the PD-related attenuation of N_e_/ERN amplitudes. The proposed inverted U-shaped relationship between dopamine levels and motor/cognitive functions suggests that reduced N_e_/ERN amplitudes in unmedicated PD patients may result from the disease-related dopamine depletion. However, the disease-related dopamine depletion, which is widely considered to be the explanation for PD-related N_e_/ERN amplitude reductions, does not predominantly affect the dopaminergic pathways that are presumably involved in N_e_/ERN generation. Therefore, it remains to be clarified whether N_e_/ERN amplitude attenuation in unmedicated PD patients is attributable to more unspecific effects of the disease (such as general disease and/or symptom burden, compatible with the results of our exploratory correlation analyses, see below), and which other mechanisms (e.g., disease-related changes in other neurotransmitter systems) contribute to this observation.

Response-locked ERP amplitudes in the latency range of N_e_/ERN have also been related to processes beyond performance monitoring. Specifically, N_e_/ERN amplitudes appear to be larger when the level of (pre-response) conflict, which is induced by the discrepancy between flanker and target arrows, is low^41^. Hence, the overall reduction of response-locked ERP amplitudes in PD compared to HC and in the medicated compared to the unmedicated state might be partly explained by changes in conflict monitoring that are related to PD and/or the activity of the dopamine system. Future studies should therefore address the role of conflict monitoring by assessing ERPs across several levels of conflict.

Exploratory correlation analyses indicated relations between N_e_/ERN amplitudes and disease duration as well as some clinical and psychological scores in PD patients. N_e_/ERN amplitudes appear to be lower in generally more impaired individuals who report higher psychological symptom burden (e.g., with regard to apathy, depression, and impulse control disorders), lower health-related quality of life (as indicated by the SF-36), and had a longer disease duration. However, conclusions from these analyses need to be drawn with caution due to their exploratory character and the small sample size. Future research may further examine how N_e_/ERN amplitudes relate to the clinical and psychological profile of PD patients and determine its usefulness as a marker, e.g., for disease progression or for the development of non-motor symptoms, such as depression or executive dysfunction. ERP measures have already been proposed to be promising in this respect, both in Parkinson’s disease^42^,^43^ and in other neurological disorders^44^,^45^,^46^,^47^,^48^, where motor symptoms are likely to distort the results of other imaging techniques or neuropsychological testing.

Similar to previous studies (e.g., refs 13, 49, 50, and 51), PD patients committed more errors on the flanker task than controls. The proportion of errors has been found to be inversely related to the magnitude of the N_e_/ERN amplitude—possibly reflecting habituation to errors^52^. Therefore, it cannot be fully excluded that the PD-related N_e_/ERN amplitude attenuation observed here results from the fact that the PD patients experienced errors more frequently than controls. However, we also observed a difference in N_e_/ERN amplitudes between the two medication conditions in the absence of medication-related differences in error rates. Hence, N_e_/ERN amplitudes did not exclusively vary with error rates in our study. Given this pattern of observations, it seems unlikely that the PD-related attenuation of N_e_/ERN amplitudes results merely from a higher frequency of erroneous responses in PD patients than in controls. The exclusion of participants due to insufficient error rates should be avoided in future studies to prevent an overrepresentation of highly error-prone individuals who may have habituated to erroneous responding. This can be achieved by increasing the overall error rates, for instance by implementing response-time pressure^53^.

We have quantified the amplitudes of N_e_/ERN and N_c_/CRN as peak amplitudes. It is, however, also possible to operationalize them by subtracting the peak of the N_e_/ERN (or N_c_/CRN) from the peak of the preceding positivity. In our study, these peak-to-peak amplitudes of N_c_/CRN and N_e_/ERN were highly correlated with the corresponding peak amplitudes (all *r*_*s*_ > 0.89, all *p* < 0.001). When we repeated our analyses with peak-to-peak amplitudes, we found a similar pattern of results, with main effects of Correctness (*F*(1, 24) = 34.39, *p* < 0.001, 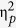 = 0.59) and Group (*F*(1, 24) = 8.13, *p* = 0.009, 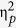 = 0.25). The Session by Group interaction showed a trend toward significance (*F*(1, 24) = 4.17, *p* = 0.052, 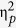 = 0.15). Hence, the core pattern of results seems to be largely unaffected by the choice of the outcome measure.

In our study, eight out of 13 patients have been examined first on their dopaminergic medication. This implies that there was a majority of PD patients who were examined off medication in their second session. Previous work has found an increase in N_e_/ERN amplitudes on a second compared to a first time of measuring^54^; this was, however, not the case in another study^55^. Here, we have observed reduced N_e_/ERN amplitudes in the on-medication state in PD patients. It is possible that this pattern of results has emerged as a consequence of the slightly unbalanced design. However, if the observed session effect on N_e_/ERN amplitude found here was a mere consequence of session order effects, a similar effect should have been present in the control participants. As indicated by a significant interaction of Group and Session, the difference between the two testing sessions differed between the PD and HC group. This latter group was composed to be imbalanced in the same manner (i.e., eight HCs were assigned to the “first session on—second session off” order and five HCs were assigned to the “first session off—second session on” order, without actually receiving dopaminergic medication at any time). Still, replication in a larger sample and with balanced order of medication states is warranted. This would also allow for further examining the error-specificity of medication-related amplitude reduction in PD patients that was not significant in our study.

Previous pharmacological studies in healthy volunteers (see refs 20 and 33 for review) have reported attenuated N_e_/ERN amplitudes following administration of the dopamine receptor antagonist haloperidol^56^,^57^, while selective depletion of the dopamine precursors phenylalanine and tyrosine did not alter N_e_/ERN amplitudes^58^. Enhanced N_e_/ERN amplitudes have been found after administration of the dopamine (and noradrenaline) agonists D-amphetamine^59^ and methylphenidate^60^. Furthermore, manipulation of prefrontal dopamine levels with sulpiride (a selective D2-receptor antagonist) revealed a U-shaped (rather than an inverted U-shaped) relationship between dopamine levels and N_e_/ERN amplitudes^61^. To our knowledge, no study has yet directly investigated how N_e_/ERN amplitudes in healthy volunteers are influenced by levodopa or dopamine agonists that are typically used as antiparkinsonian medication. However, one study has reported increased amplitudes of the feedback-related negativity (FRN)—an ERP waveform that is closely related to the N_e_/ERN^5^—after a single dose of pramipexole, a dopamine agonist for the treatment of PD^62^. In contrast to these data, our results suggest that dopaminergic medication in PD patients is related to a reduction rather than to an enhancement of N_e_/ERN amplitudes. At this point, it remains unclear whether this pattern of evidence may be attributable to the different pharmacological characteristics of dopaminergic medication typically administered to PD patients and the dopaminergic drugs that were related to increased N_e_/ERN amplitudes in refs 59, 60, and 61, or whether it reflects that the effects of dopaminergic drugs are not comparable between PD patients and healthy individuals. Placebo-controlled studies with levodopa and dopamine agonists (that are used as antiparkinsonian medication) comparing healthy volunteers and PD patients are needed to determine (a) whether the described discrepancies may be explained by the different pharmacological profiles of the dopaminergic agents, and (b) how the effects of dopaminergic agents differ between PD patients and healthy individuals.

The data presented here are partly consistent with earlier research reporting reduced N_e_/ERN amplitudes in patients with PD (however, the PD-related ERP amplitude reduction was not specific to errors in the present study). The effect of dopaminergic medication on response-locked ERP amplitudes supports the hypothesis that N_e_/ERN amplitude attenuation found in PD might be dopamine-dependent. We conclude that the N_e_/ERN amplitude appears to be sensitive to dopamine overdosing that potentially occurs as a corollary of the dopaminergic treatment of motor symptoms in PD.

## Additional Information

**How to cite this article:** Seer, C. *et al*. Dopaminergic modulation of performance monitoring in Parkinson’s disease: An event-related potential study. *Sci. Rep.* **7**, 41222; doi: 10.1038/srep41222 (2017).

**Publisher's note:** Springer Nature remains neutral with regard to jurisdictional claims in published maps and institutional affiliations.

## Supplementary Material

Supplementary Material

## Figures and Tables

**Table 1:** Means (M) and standard deviations (SD) of demographic, clinical and psychological characteristics of the patients with Parkinson’s disease (PD) and healthy controls (HC).

measure	max	HC (*N* = 13)		PD (*N* = 13)	
		*M*	*SD*	*M*	*SD*
age [years]		63.15	11.15	64.31	8.66
education [years]		13.65	3.24	13.04^a^	3.82
MoCA (cognitive status)	30	28.23	1.83	26.38	1.76
WST (premorbid intelligence)	42	29.69	4.03	29.15	4.41
AES (apathy)	54	10.23	7.94	16.15	6.62
BDI-II (depression)	63	6.31	6.36	8.85	7.56
BSI-18 (psychiatric status)	72	5.25^a^	5.74	11.27^b^	10.84
anxiety	24	1.50^a^	1.62	4.09^b^	3.70
depression	24	1.67^a^	2.19	3.55^b^	4.76
somatization	24	2.08^a^	2.75	3.64^b^	3.47
SF-36 (health status)	100	72.34^a^	21.15	56.59^b^	22.25
physical functioning	100	76.67^a^	26.14	45.91^b^	31.53
physical role functioning	100	56.25^a^	47.82	47.73^b^	39.46
bodily pain	100	72.42^a^	25.05	57.00^b^	36.21
general health perception	100	62.25^a^	25.35	51.09^b^	23.11
vitality	100	65.00^a^	21.74	52.73^b^	19.67
social role functioning	100	86.46^a^	15.50	69.32^b^	20.44
emotional role functioning	100	83.33^a^	33.33	69.70^b^	45.84
mental health	100	76.33^a^	16.22	59.27^b^	22.19
BIS-Brief (impulsiveness)	32	14.62	3.31	15.03	4.06
DII (impulsivity)					
functional	11	6.15	3.00	5.23	2.55
dysfunctional	12	2.15	2.19	3.00	3.65
QUIP-RS (impulse control)	112	0.09^b^	0.30	9.83^a^	11.10
impulse control disorder	64	0.08	0.28	5.33^a^	5.63
SPQ (schizotypal traits)	22	5.33^a^	4.42	5.83^a^	4.93
interpersonal	8	3.25^a^	3.02	2.75^a^	2.26
cognitive-perceptual	8	1.00^a^	0.85	1.33^a^	1.30
disorganized	6	1.08^a^	1.24	1.75^a^	2.30

Note. max = maximal value of the respective measure; MoCA = Montreal Cognitive Assessment^22^; WST = Wortschatztest^27^; AES = Apathy Evaluation Scale^63^; BDI-II = Beck Depression Inventory-II^64^; BSI-18 = Brief Symptom Inventory (18-item version)^65^; SF-36 = Short Form Health Survey^66^; BIS-Brief = Barratt Impulsiveness Scale (8-item version)^67^; DII = Dickman Impulsivity Inventory^68^; QUIP-RS = Questionnaire for Impulsive-Compulsive Disorders in Parkinson’s Disease—Rating Scale^69^; SPQ = Schizotypal Personality Questionnaire^70^.

^a^Based on *n* = 12.

^b^Based on *n* = 11.

**Table 2:** Usual daily dose of antiparkinsonian medication (in mg/d) administered to the patients with Parkinson’s disease (PD) at the time of the examination “on” medication and individual levodopa equivalent daily doses (LEDD; in mg/d).

Case	Medication	LEDD
1	Pramipexole 3.15^a^	450
2	L-Dopa 550, L-Dopa^b^ 300, Tolcapone 300, Pramipexole 2.45^a^, Amantadine 400	1,800
3	L-Dopa 300, L-Dopa^b^ 100, Pramipexole 3.15^a^	825
4	L-Dopa 125, L-Dopa^b^ 500, Pramipexole 2.1^a^, Amantadine 600	1,400
5	L-Dopa 300, Entacapone 600	399
6	L-Dopa 400, L-Dopa^b^ 100, Rasagiline 1	575
7	L-Dopa 1,000, Entacapone 1,000, Pramipexole 1.75^a^, Selegiline 10^c^	1,680
8	L-Dopa 400	400
9	L-Dopa 600, Entacapone 600, Pramipexole 1.04^a^	948
10	L-Dopa 600	600
11	L-Dopa 600, Rotigotine 6, Cabergoline 6, Amantadine 200	1,380
12	L-Dopa 600, L-Dopa^b^ 300, Entacapone 800, Cabergoline 6	1,497
13	L-Dopa 700, L-Dopa^b^ 100, Entacapone 600, Rotigotine 8	1,246

Note. ^a^The conversion factor for Pramipexole provided by ref. 24 refers to the salt form of Pramipexole (Pramipexole dihydrochloride 1 H_2_O). We used the dose of the Pramipexole salt form to calculate the LEDD, but report the corresponding dose of Pramipexole (base form) here for easier interpretation. For example, Pramipexole 3.15 (base form) corresponds to 4.5 mg Pramipexole dihydrochloride 1 H_2_O (salt form). ^b^Controlled-release L-Dopa dose. ^c^Oral Selegiline.

**Table 3:** Means (M) and standard deviations (SD) of median reaction times (RT) and error rates (ER) for patients with Parkinson’s Disease (PD) and healthy controls (HC) for the two sessions (off, on) and as a function of stimulus congruency (congruent, incongruent).

		HC (*N* = 13)		PD (*N* = 13)	
		*M*	*SD*	*M*	*SD*
RT (ms) (correctly completed trials)					
off	congruent	406	60	414	77
	incongruent	493	82	494	89
on	congruent	412	57	466	162
	incongruent	494	62	529	146
RT (ms) (error trials)					
off	congruent	491^a^	198^a^	462	163
	incongruent	392	157	369	67
on	congruent	433^a^	140^a^	467	186
	incongruent	373	133	451	220
ER (%)					
off	congruent	1.5	1.4	8.8	7.0
	incongruent	10.4	5.5	21.8	19.2
on	congruent	1.5	1.5	7.5	5.9
	incongruent	10.4	5.6	20.8	14.4

Note. For PD patients, the conditions “off” and “on” indicate the actual medication state (i.e., whether the patients had taken their usual dose of medication [on] or were tested after withdrawal from dopaminergic medication [off]). The HC group was not administered with dopaminergic medication at any time, and the conditions “off” and “on” were merely used to assign control participants to one of two possible orders of medication conditions (see Methods for detailed explanation).

^a^Based on *n* = 11.

**Table 4:** Spearman-Brown rank correlations between N_e_/ERN amplitudes and clinical/psychological characteristics in patients with Parkinson’s disease (PD) and healthy controls (HC).

	HC (*N* = 13)		PD (*N* = 13)	
	off	on	off	on
disease duration	—	—	0.609*	0.160
LEDD	—	—	0.220	0.148
age	0.105	0.074	0.609*	0.412
education	−0.055	0.114	−0.391	−0.173
cognitive status (MoCA)	−0.495	−0.225	−0.155	−0.293
apathy (AES)	−**0.207**	−**0.190**	0.675*	0.694**
depression (BDI-II)	**−0.504**	−0.342	0.623*	0.421
psychiatric status (BSI-18)	−0.**243**	−0.047	0.743**	0.532
health status (SF-36)	**0.315**	−0.063	−0.764**	−0.409
impulsiveness (BIS-Brief)	0.003	−0.136	0.556*	0.415
functional impulsivity (DII functional)	0.192	0.070	−0.502	−0.161
dysfunctional impulsivity (DII dysfunctional)	−0.114	−0.342	0.469	0.363
impulse control (QUIP-RS)	0.400	0.500	0.573	0.406
impulse control (QUIP-RS impulse control disorder)	0.386	0.463	0.602*	0.384
schizotypal traits (SPQ)	−**0.250**	0.071	0.631*	0.222

Note. Positive correlations indicate that higher scores on the respective measure are associated with less negative (i.e., smaller) N_e_/ERN amplitudes. Negative correlations indicate that higher scores on the respective measure are associated with more negative (i.e., larger) N_e_/ERN amplitudes. Bold typeface indicates significant differences of correlation coefficients between PD and HC (*p* < 0.05). For PD patients, the conditions “off” and “on” indicate the actual medication state (i.e., whether the patients had taken their usual dose of medication [on] or were tested after withdrawal from dopaminergic medication [off]). The HC group was not administered with dopaminergic medication at any time, and the conditions “off” and “on” were merely used to assign control participants to one of two possible orders of medication conditions (see Methods for detailed explanation). LEDD = levodopa equivalent daily dose, calculated using the conversion factors by ref. 24; MoCA = Montreal Cognitive Assessment^22^; WST = Wortschatztest^27^; AES = Apathy Evaluation Scale^63^; BDI-II = Beck Depression Inventory-II^64^; BSI-18 = Brief Symptom Inventory (18-item version)^65^; SF-36 = Short Form Health Survey^66^; BIS-Brief = Barratt Impulsiveness Scale (8-item version)^67^; DII = Dickman Impulsivity Inventory^68^; QUIP-RS = Questionnaire for Impulsive-Compulsive Disorders in Parkinson’s Disease—Rating Scale^69^; SPQ = Schizotypal Personality Questionnaire^70^.

*p < 0.05, **p < 0.01.
