# Supplementary material for: Dopaminergic modulation of performance monitoring in Parkinson’s disease: An event-related potential study
Source: Sci Rep. 2017 Jan 24;7:41222. doi: 10.1038/srep41222 (PMC5259704; doi:10.1038/srep41222)
Supplement: Supplementary Material [file srep41222-s1.pdf]

Dopaminergic modulation of performance monitoring in Parkinson's disease:

An event-related potential study

Caroline Seer, Florian Lange, Sebastian Loens, Florian Wegner, Christoph Schrader, Dirk Dressler, Reinhard Dengler, Bruno Kopp

Supplementary Material

Table S1

Means (M), standard deviations (SD), and range (min, max) of the numbers of trials included in the analysis of response-locked ERPs (N_c/CRN, N_e/ERN) in the two medication conditions for patients with Parkinson's disease (PD) and healthy controls (HC).

		HC (N = 13)				PD (N = 13)			
		M	*SD*	min	max	*M*	*SD*	min	max
N_c/CRN	off	193	12	167	204	169	36	88	205
	on	192	14	153	204	171	29	114	207
N_e/ERN	off	22	12	9	46	46	37	10	127
	on	22	13	12	59	43	27	9	96

Note. For PD patients, the conditions "off" and "on" indicate the actual medication state (i.e., whether the patients had taken their usual dose of medication [on] or were tested after withdrawal from dopaminergic medication [off]). The HC group was not administered with dopaminergic medication at any time, and the conditions "off" and "on" were merely used to assign control participants to one of two possible orders of medication conditions (see Methods for detailed explanation).

Table S2

Means (M) and standard deviations (SD) of response-locked ERP amplitudes (in µV) for patients with Parkinson's disease (PD) and healthy controls (HC).

	HC (*N* = 13)		PD (*N* = 13)	
	M	*SD*	*M*	*SD*
off Session				
N_c/CRN amplitude	-2.06	2.25	-1.10	1.88
N_e/ERN amplitude	-5.01	2.92	-3.26	1.82
total amplitude	-3.54	2.15	-2.18	1.27
on Session				
N_c/CRN amplitude	-2.55	1.32	-0.92	1.76
N_e/ERN amplitude	-5.19	3.05	-2.33	1.61
total amplitude	-3.87	2.02	-1.62	1.20
averaged across off and on Sessions				
N_c/CRN amplitude	-2.31	1.69	-1.01	1.76
N_e/ERN amplitude	-5.10	2.85	-2.79	1.61
total amplitude	-3.70	2.04	-1.90	1.16

Note. For PD patients, the conditions "off" and "on" indicate the actual medication state (i.e., whether the patients had taken their usual dose of medication [on] or were tested after withdrawal from dopaminergic medication [off]). The HC group was not administered with dopaminergic medication at any time, and the conditions "off" and "on" were merely used to assign control participants to one of two possible orders of medication conditions (see Methods for detailed explanation).

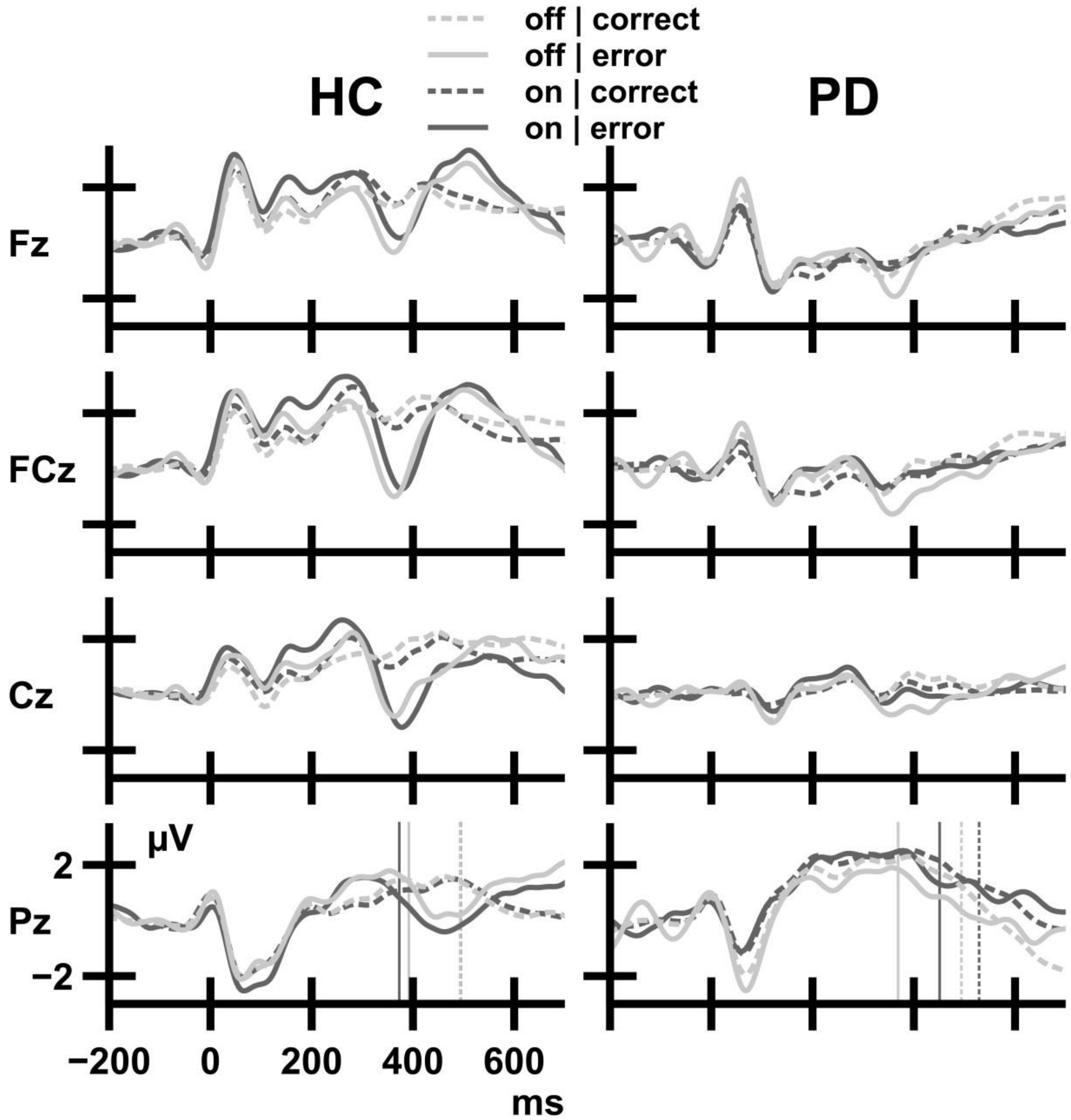

Figure S1. Grand average ERP waveforms at midline electrodes for healthy controls (HC, left panels) and patients with Parkinson's disease (PD, right panels). ERPs are synchronized to incongruent stimuli, 0 ms indicates the time of target onset. Vertical lines in the bottom panels indicate the average reaction time (RT) per group and condition. Note that for HC, correct RTs for the two session conditions are virtually identical (off: 493 ms, on: 494 ms). For PD patients, the conditions "off" and "on" indicate the actual medication state (i.e., whether the patients had taken their usual dose of medication [on] or were tested after withdrawal from dopaminergic medication [off]). The HC group was not administered with dopaminergic medication at any time, and the conditions "off" and "on" were merely used to assign control participants to one of two possible orders of medication conditions (see Methods for detailed explanation).
